# Anatomy and Pathologies of the Spinous Process

**DOI:** 10.3390/diseases12120302

**Published:** 2024-11-26

**Authors:** Sisith Ariyaratne, Nathan Jenko, Karthikeyan P. Iyengar, Mark Davies, Christine Azzopardi, Simon Hughes, Rajesh Botchu

**Affiliations:** 1 Department of Musculoskeletal Radiology, Royal Orthopedic Hospital, Birmingham B31 2AP, UK; sisith.ariyaratne@nhs.net (S.A.); n@jenko.eu (N.J.); mark.davies8@nhs.net (M.D.); christine.azzopardi1@nhs.net (C.A.); 2 Department of Orthopedics, Southport and Ormskirk Hospitals, Mersey and West Lancashire Teaching NHS Trust, Southport PR8 6PN, UK; kartik.iyengar@merseywestlancs.nhs.uk; 3 Apollo Hospitals Educational & Research Foundation (AHERF), New Delhi 11007, India; 4 Edge Hill University, Ormskirk L39 4QP, UK; 5 Department of Spinal Surgery, The Royal Orthopaedic Hospital, Birmingham B31 2AP, UK; simon.hughes9@nhs.net; 6 Department of Radiology, NRI Institute of Medical Sciences, Visakhapatnam 531163, India

**Keywords:** spinous process, neuroimaging, computed tomography, magnetic resonance imaging, neoplasm, congenital, baastrup disease

## Abstract

The spinous processes act as a lever for attachments of muscles and ligaments. Spinal imaging is commonly performed as a diagnostic test for pain and radiculopathy. A myriad of incidental or unexpected findings, both potentially asymptomatic and symptomatic, may be encountered during the interpretation of these images, which commonly comprise radiographs, Computed Tomography (CT) and Magnetic Resonance Imaging (MRI). Isolated lesions of the spinous process, although less common, are some of the lesions that may be encountered and can present a diagnostic dilemma. These can range from congenital abnormalities, traumatic lesions, neoplasms and lesions of inflammatory, infective and metabolic aetiology. The literature specifically reviewing these lesions is sparse. The article reviews a range of pathologies affecting the spinous process, along with their pertinent imaging features, based on isolated pathologies of spinous process lesions identified on imaging by the authors at a tertiary orthopaedic centre over a 10-year period. A search on the hospital Picture Archive and Communication System (PACS) and Radiology Information System (RIS) was performed using the keyword “spinous process” and a list of the isolated pathologies of the spinous process based on the imaging reports was compiled for the purpose of this narrative review. It is important that radiologists consider these lesions when they are identified on routine imaging of the spine.

## 1. Introduction

Spinal imaging is a commonly performed investigation, often as a first line for pain and radiculopathy. Isolated lesions of the spinous process may be encountered on imaging, and these include neoplasms, congenital lesions, infective and inflammatory pathologies and pathologies caused by trauma. A significant proportion of these can be asymptomatic and thus incidentally identified [1]. While isolated spinous process lesions are less common, they can present a diagnostic dilemma and may require further characterisation and work-up.

Commonly used imaging modalities for further characterisation of the lesions include Magnetic Resonance Imaging (MRI) and Computed Tomography (CT) [2,3]. Radionuclide (bone) scans and 18-FDG-PET (Fluorodeoxyglucose Positron Emission Tomography) scans are useful with neoplastic lesions, and when multiple lesions are involved. While conventional radiography is a useful preliminary imaging tool, it may have limited value in the characterisation of the pathologies involved [2,3].

The literature specifically reviewing commonly encountered incidental, isolated lesions of the spinous processes is sparse, and awareness of these pathologies and relevant features is vital for radiologists involved in routine interpretation of spinal imaging. This narrative review article reviews the commonly encountered lesions of the spinous process, based on a retrospective review of such cases at a tertiary orthopaedic centre over a 10-year period.

## 2. Anatomy

Spinous processes are bony projections arising from the posterior aspect of cervical, thoracic and lumbar vertebrae at the junction of the laminae [4]. They serve an important role in the structural integrity of the spine and form vital attachment points for muscles (erector spinae and transversospinalis muscle groups) and ligaments, which mainly include the interspinous and thoracolumbar supraspinous ligaments, and the nuchal ligament in the cervical spine [4].

Their morphology varies based on the type of vertebra; as a general rule, the cervical spinous processes are shorter, angled inferiorly and tend to be bifid, the thoracic spinous processes are longer, slender and angulated inferiorly to accommodate rib articulations, and the lumbar spinous processes are larger, broader and more horizontally oriented [5,6]. Two common exceptions to this principle are C1, which lacks a distinct spinous process, C2, which has a larger spinous process compared to other cervical vertebrae and C7, which has a longer and more distinct spinous process that is not bifid but rather terminates in a round tubercle [5]. Spinous processes can also provide useful anatomical landmarks during clinical examination and can be used to guide percutaneous procedures.

## 3. Pathologies Affecting the Spinous Process

### 3.1. Congenital

#### 3.1.1. Hyperplasia and Hypoplasia

These are rare abnormalities and are often incidentally identified. In the case of hyperplasia, patients may also describe a painless swelling in the posterior midline, and the entity is most commonly encountered in the cervical spine. Imaging may reveal an abnormally enlarged spinous process. It can be unilateral, affecting one of the bifid components of the cervical spinous processes, and may involve the lamina. They may increase in size with growth in the paediatric population [7]. The finding may be associated with other vertebral anomalies such as fusion defects, hemivertebrae and scoliosis [7], and one must carefully assess for the presence of these associated pathologies when spinous process hyperplasia is encountered. Hyperplasia of the C2 spinous process in particular is associated with congenital absence of the posterior arch of C1 [8].

Hypoplasia represents a congenitally malformed and small spinous process. [9] Isolated hypoplasia can be a cause of mechanical back pain [10].

#### 3.1.2. Fusion Abnormalities

A part of the spectrum of pathologies associated with spinal dysraphism, fusion defects of the posterior neural arch can give rise to an unfused spinous process resulting in a cleft [11]. This may also be associated with central nervous system abnormalities. With milder forms, termed spina bifida occulta, it is often incidentally identified. The lumbar vertebrae are usually involved. The adjacent lamina can be involved, in which case the spinous process can remain unattached, held by the ligamentum flavum or, alternatively, may fuse with an adjacent spinous process [9].

#### 3.1.3. Apophysis

Each vertebra consists of an ossification centre, which ossifies to form the neural arch within the first 3 to 5 years. After puberty, an accessory ossification centre (apophysis) is formed at the tip of the spinous process, which only fuses by the age of 25 [12]. Occasionally, these centres do not fuse, resulting in a smooth-appearing non-united ossicle, without disproportionate sclerosis [13].

#### 3.1.4. Other Normal Variants

While not pathological lesions, a range of normal variants are also encountered in the spinous process, which can mimic pathology and should not be mistaken for such. These include anomalous articulations and ossicles between spinous processes, spinous process fissures which can mimic a fracture, and abnormal vertical orientation of a bifid cervical spinous process [8].

### 3.2. Degenerative/Inflammatory

#### Baastrup Disease

Usually seen in the lumbar spine and a cause of back pain, Baastrup disease is a result of degenerative hypertrophy, inflammatory change and bursa formation between two adjacent spinous processes. Excessive lordosis is a contributing factor.

**Figure 1:**
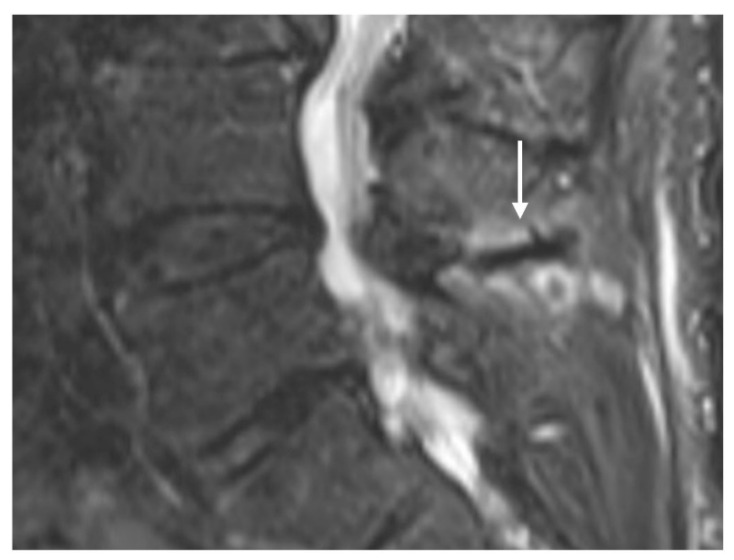
Sagittal STIR (short tau inversion recovery) image demonstrating Baastrup disease of lumber spinous processes (white arrow). Note the presence of reactive marrow oedema and sclerosis at the opposing surfaces of the spinous processes involved.

Characteristic imaging features include a close approximation of lumbar spinous processes (‘kissing spines”). CT may show reactive sclerosis, the resultant enlargement of the abutting spinous processes, and pseudoarthrosis formation. MRI can demonstrate interspinous bursal fluid as well as marrow oedema (Figure 1) [14]. Large bursal fluid collections can extend into the vertebral canal causing thecal sac compression.

### 3.3. Neoplastic

#### 3.3.1. Osteoid Osteoma

**Figure 2:**
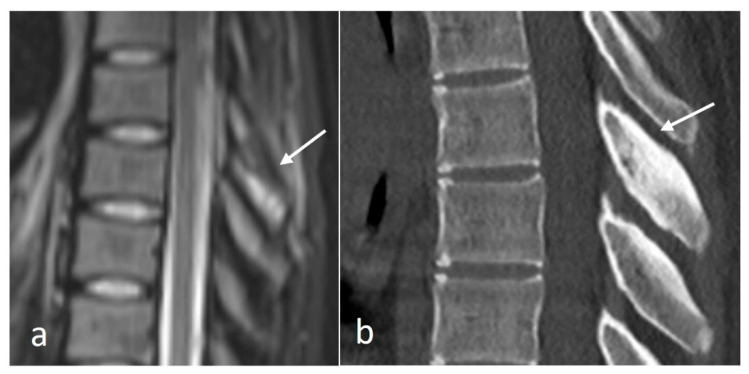
Sagittal STIR (**a**) and CT (**b**) images demonstrating a thoracic spinous process osteoid osteoma (white arrows). Note the presence of reactive marrow oedema on the STIR sequence. The sclerotic reactive bone is well delineated on CT, although the lucent nidus may not always be present as in this case.

Osteoid osteomas (OOs) account for 10–14% of primary vertebral tumours. The majority are seen in the lumbar spine, followed by the cervical and thoracic spine. Given their predilection for the posterior elements, involvement of the spinous process is not uncommon [15,16]. MRI is sensitive and may show marrow oedema; however, the oedema can obscure the nidus, which can be a potential pitfall. On MRI, the nidus when seen is of low to intermediate T1 signal, ofvariable T2 signal with areas of signal void due to mineralisation (Figure 2a). The nidus may also show variable enhancement on post-contrast imaging [15,16]. Lesions may be occult on a radiograph. The presence of sclerotic reactive bone surrounding a lucent nidus is a typical feature on CT, although the latter may not always be present (Figure 2b). A central sclerotic dot may also be present. The nidus is typically <1.5 to 2 cm in diameter.

#### 3.3.2. Osteochondroma

**Figure 3:**
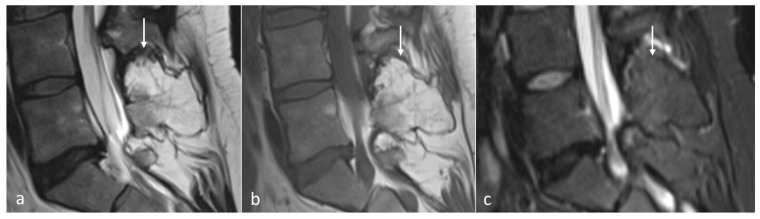
Sagittal T2 (**a**), T1 (**b**) and STIR (**c**) images showing an osteochondroma of the L5 spinous process (white arrows). Note the presence of the thin cartilage cap on its superior aspect.

Osteochondroma is a cartilage-capped bony exostosis and the commonest benign bone lesion. They occur spontaneously or in the setting of Hereditary Multiple Exostosis (HME). The majority of spinal osteochondromas occur in the cervical spine [17]. The morphology can be either sessile or pedunculated, and they have a cartilage cap, which is best appreciated on MRI (Figure 3a–c). The cartilage cap usually demonstrates high T2 signal and should be <15 mm in diameter. Increased thickness of the cap can be a sign of malignant degeneration [18].

#### 3.3.3. Giant Cell Tumour (GCT)

GCTs are the most aggressive primary benign tumour of the spine, with a high recurrence rate. As a result, they can often be accompanied by pain and neurological symptoms. A small subset of GCTs can be malignant, with similar imaging features across the two groups [15,18].

On CT, GCT usually appears as a lytic expansile lesion with a thin peripheral shell of bone. The lesions are usually mixed cystic and solid, a feature best appreciated on MRI. The tumour itself may show intermediate to low signal intensity on both T1 and T2. Secondary aneurysmal bone cyst change is also a common feature, with fluid–fluid levels present on MRI. The soft tissue components are also best appreciated on MRI and tend to enhance. Perilesional oedema and extra-osseous soft tissue components also can be seen [19].

#### 3.3.4. Aneurysmal Bone Cyst (ABC)

**Figure 4:**
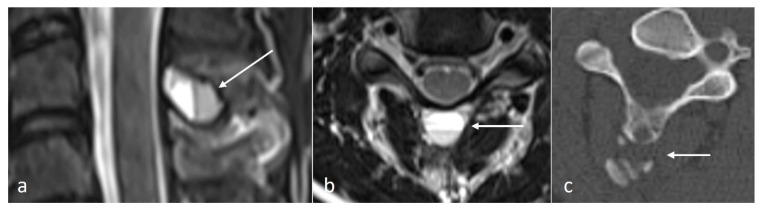
Sagittal T2 (**a**), axial T2 (**b**) and axial CT (**c**) images demonstrating an aneurysmal bone cyst of a cervical spinous process (white arrows). Note the lytic osseous destruction on CT and the characteristic presence of fluid–fluid levels on MRI.

ABCs are benign locally aggressive lesions, with a predilection for the posterior spinal elements [15,18]. The lesions tend to be lytic and expansile. Thin bony septa may be present within the lesion, best appreciated on CT. MRI will show a cystic lesion with high signal on fluid-sensitive sequences, and the typical fluid–fluid levels. (Figure 4a–c) Rarely, solid components may also be present. These components as well as the septa can enhance.

It is important to distinguish primary ABCs from secondary ABC change occurring in association with other lesions such as fibrous dysplasia, GCT and even osteosarcoma, and the rare telangiectatic osteosarcoma, although osteosarcomas isolated to the spinous process are rare [3].

#### 3.3.5. Haemangioma

These are the commonest primary benign tumours of the spine and are frequently encountered incidentally. They usually occur in the vertebral body, and, hence, involvement of the spinous process is rare. The vast majority (99%) are asymptomatic, and the symptomatic ones tend to more commonly involve the posterior elements or be aggressive haemangiomas [3]. They are hamartomatous lesions, containing vessels interspersed within trabecular bone and typically also contain fat [15].

**Figure 5:**
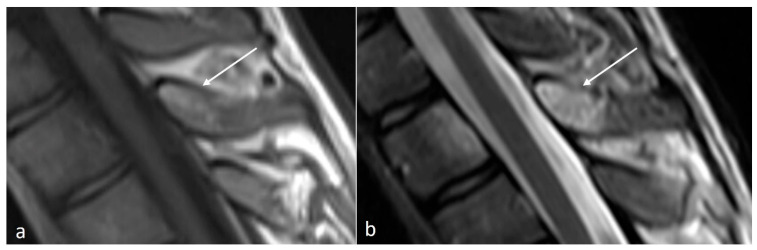
Sagittal T1 (**a**) and T2 (**b**) MRI images demonstrating a thoracic spinous process haemangioma (white arrows). Note the presence of high T1 and T2 signals, which are characteristic of a typical haemangioma due to the fat content.

CT typically shows trabecular bone interwoven with fatty marrow, and the ‘Corduroy’ and ‘polka dot’ signs, although the latter may not be seen in those involving the spinous process. On MRI, they tend to be hyperintense to marrow on both T1 and T2 due to high fat content (Figure 5a,b) and can show >20% signal intensity drop on opposed phase chemical shift imaging [18]. Aggressive haemangiomas can lack these typical features and as such may demonstrate bone destruction and extra-osseous soft tissue components [15,18].

#### 3.3.6. Fibrous Dysplasia (FD)

FD is a benign lesion characterised by intramedullary fibro-osseous proliferation due to altered osteogenesis [20]. It can be monostotic or polyostotic and can also occur in association with several syndromes [15]. Its occurrence is rare in the spine, and when spinal involvement is present, the disease is more likely to be polyostotic.

CT typically demonstrates a typical “ground-glass” matrix, with a variable degree of intralesional ossification and cystic change. The lesions can also be lytic and expansile with a thin peripheral cortical rim [18]. On MRI, the lesions are low on T1 and intermediate to high signal on fluid-sensitive sequences. Secondary ABC change can also be present [18].

#### 3.3.7. Chondroblastoma

Chrondroblastoma is a benign cartilaginous lesion, which is rare in the spine [21]. They typically affect the younger population, with a mean age of diagnosis of 31.9 years [22].

On CT, they typically appear as osteolytic lesions, and, as such, may be inseparable from other lesions. A variable degree of intralesional calcification (chondroid matrix) may also be present, which, if seen, would favour a cartilaginous tumour [21]. Similar features can be seen with chondrosarcoma, and the latter should be considered in the differential diagnosis if these features are present. An extra-osseous soft tissue component also can be present, better delineated on MRI. Lesions demonstrate enhancement on post-contrast imaging.

#### 3.3.8. Metastases

These are the commonest malignant tumours of the spine, comprising approximately 90% of masses encountered on spinal imaging according to one study [23]. These represent the spread of primary tumours of the spine and are an important differential to consider when spinous process lesions are encountered. The spine is the commonest site of osseous metastasis, with spinal metastases present in 5–10% of cancer patients, and while the presence of multiple lesions often provides a diagnostic clue [24,25], isolated lesions may present a diagnostic dilemma. The majority of lesions tend to be symptomatic, with pain being the commonest symptom.

Metastases can either be osteolytic or osteoblastic [26]. Lytic metastases are typically hypodense on CT, with variable degrees of osseous destruction, and osteoblastic (sclerotic) metastases appear hyperdense. On MRI, lytic metastases are typically of intermediate signal on T1 sequences, and iso- to hyperintense on fluid-sensitive sequences [18]. Sclerotic metastases show reduced signal intensity on both T1 and fluid-sensitive sequences, and may only be seen on fat-suppressed sequences by a halo of surrounding marrow oedema [18]. Mixed lytic and sclerotic metastatic lesions are also common, and melanoma metastases can be hyperintense on T1 [18].

#### 3.3.9. Multiple Myeloma and Plasmacytoma

Multiple myeloma (MM) is a neoplastic proliferation of plasma cells, which can affect multiple organs, and osseous involvement is common [27]. The lesions often tend to be multiple, hence, solitary involvement of a spinous process without involvement of the other visualised bones would be uncommon. The spectrum of disease also includes plasmacytoma, which tends to be solitary.

**Figure 6:**
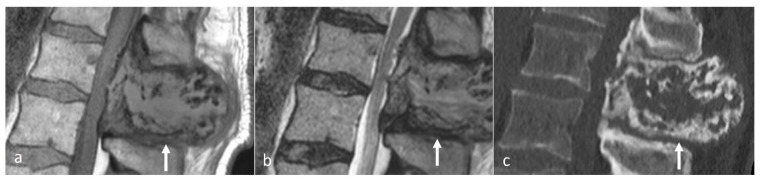
Sagittal T1 (**a**), T2 (**b**) and CT (**c**) images of a sclerotic myelomatous deposit involving a thoracic spinous process (white arrows). While myeloma typically tends to present as lytic lesions, occasionally they can be expansile and sclerotic as seen here.

The typical imaging appearance is a well-circumscribed lytic lesion. They can occasionally be expansile and also sclerotic (Figure 6a–c). On MRI, the lesions typically appear as a T1 and T2 hypointense mass, which shows high signal on T2 STIR or T2 fat-saturated images. The lesions enhance after contrast administration [27].

#### 3.3.10. Eosinophilic Granuloma (EG)

EG is a localised form of Langerhans cell histiocytosis (LCH), seen in the paediatric population usually in the first decade. They may be occasionally seen in young adults as well. While the spine is a common site of involvement, they have a predilection for the vertebral body with sparing of the posterior elements, hence, spinous process involvement is unusual. The lesions are typically painful [18].

**Figure 7:**
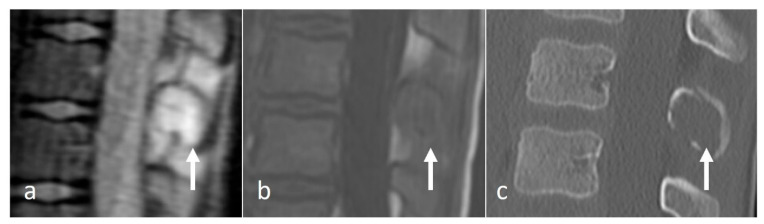
Sagittal STIR (**a**), T1 (**b**) and CT (**c**) images demonstrating an eosinophilic granuloma of a thoracic spinous process (white arrows) in a paediatric patient. The lytic appearance is typical of these lesions.

They appear as well-defined lytic lesions. Extra-osseous soft tissue components may be seen on MRI. MRI signal characteristics tend to be non-specific, with lesions being hypo- to isointense on T1 and hyperintense fluid-sensitive sequences. The lesions enhance with contrast (Figure 7a–c) [15,18].

### 3.4. Trauma

#### Fracture, Apophyseal Injury and Non-Union

The most classical fracture of the spinous processes is the inferior cervical and/or superior thoracic spinous “clay shoveler’s” fracture [28]. This is a stress fracture caused by repeated stress on the spinous process exerted by the trapezius and rhomboids. The C7 and T1 spinous processes are thought to be most vulnerable due to their length and perpendicular orientation to muscle [29,30].

**Figure 8:**
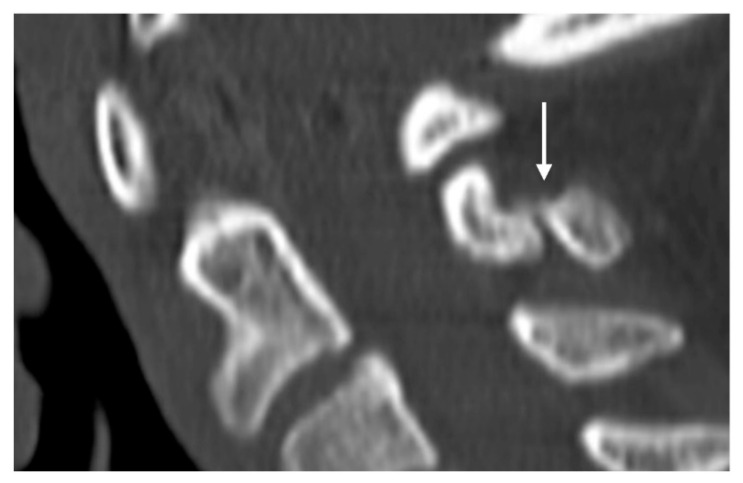
Sagittal CT image demonstrating a non-united fracture of C2 spinous process (white arrow).

These fractures can be readily visualised on a plain film or CT, where a displaced fragment without a sclerotic edge can be seen. MR demonstrates bone marrow oedema (T2 hyperintensity) at the fracture site with frequent oedema within the interspinous ligament. Chronic non-union is a complication and is characterised by sclerosis and/or irregularity of the fracture line (Figure 8) and by resolution of oedema on MRI.

Spinous fractures also occur due to direct trauma. In most cases, these would be expected to occur with other more significant spinal fractures. However, isolated traumatic spinal process fractures have been described [31,32]. Additionally, osteoporosis is a significant risk factor for spinous process fracture [33]. In the context of trauma, CT is the gold standard imaging modality.

In children, the apophysis is weaker than the interspinous ligament and can be injured. In most cases, healing will occur with rest, but the spinous process tip may appear irregular and sclerotic [34]. In athletes, repeated injury may result in non-union with a displaced fragment or pseudo-arthrosis. CT allows the delineation of the non-united fragment with sclerosis and irregularity at the fracture line. MRI demonstrates bone marrow oedema and bone scintigraphy demonstrates increased uptake [12].

### 3.5. Metabolic

#### Paget’s Disease

Paget’s disease is a common metabolic bone disease, with a possible genetic predisposition [35]. Polyostotic disease is more common than monostotic disease. The lumbar spine is one of the commonest sites involved (30–50%) with the thoracic spine being more uncommon and cervical spine involvement being rare (2–4%) [36]. Initially, the disease is asymptomatic and is often diagnosed on imaging incidentally [37].

Irrespective of location, imaging appearances are defined by the three phases of the disease. During the initial phase, lysis predominates; the lytic phase is difficult to detect on imaging in the spine due to a high trabecular/cortex ratio. The second phase involves simultaneous lysis and bone formation, but notably the deposited bone is abnormal and of reduced strength. The final blastic phase is characterised by abnormal bone deposition, which results in enlargement and sclerosis (“ivory vertebra”) [38,39].

Changes in the vertebra are characterised by endosteal resorption, which results in the expansion of the central bone marrow. Blastic activity is concentrated at the periosteum (periosteal apposition) resulting in new bone formation at the margins, thus resulting in enlargement [39]. The entire vertebra including the posterior elements is normally involved and periosteal apposition in the posterior elements will result in central canal narrowing, which can result in significant stenosis [40].

While expansion can be seen on radiographs, CT is the gold standard to demonstrate Paget’s disease as well as posterior element expansion. Bone marrow is not affected by Paget’s disease, although subtle signal changes may be present. Bone marrow replacement on MRI must raise suspicion of a separate pathological process, including metastases and, rarely, sarcomatous transformation [41].

### 3.6. Osteomyelitis

Osteomyelitis of the vertebrae occurs usually due to infection by a single pathogen, with *Staphylococcus aureus* bacterium being the commonest aetiological organism. The condition occurs primarily due to haematogenous dissemination of infection, but can also occur post-surgery and after trauma. Due to the initial insidious and non-specific clinical presentation, initial diagnosis can be challenging and delayed [42].

**Figure 9:**
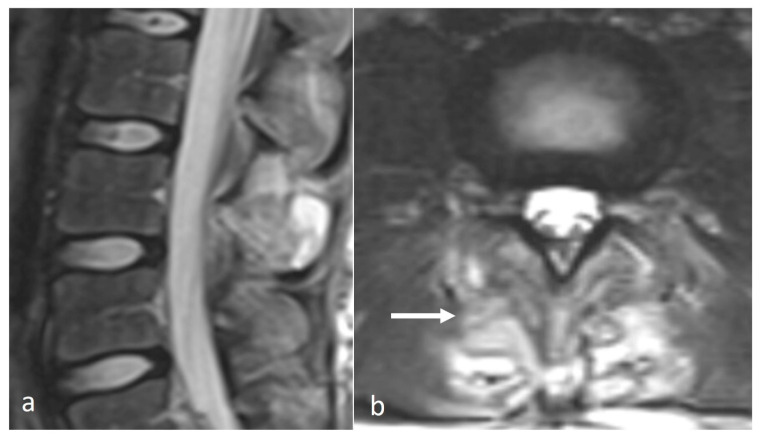
Sagittal (**a**) and axial (**b**) STIR sequences demonstrating osteomyelitis of the L4 spinous process, characterised by extensive marrow oedema (white arrow). The oedema also extends to the adjacent laminae in this case, and there is signal change in the adjacent paraspinal muscles, which is a common feature.

While the spine is a common site of infection, endplate involvement is most common, but spinous process osteomyelitis can be occasionally encountered. Correlation with accompanying clinical features and biochemical markers is crucial for accurate diagnosis. MRI is the most sensitive imaging modality, and the earliest abnormality is the presence of subchondral marrow oedema, with reduced T1 signal intensity and increased fluid signal intensity, with enhancement following contrast. This can eventually progress to osseous irregularity and erosion. Inflammatory changes in the adjacent paravertebral soft tissue are also common (Figure 9a,b) [18,42].

## 4. Conclusions

A broad range of pathologies affecting the spinous process may be encountered in routine imaging of the spine. The spinous process can be an unusual location for a significant number of these pathologies, and as such may present a diagnostic dilemma. In order to correctly identify these lesions and consider an appropriate range of differential diagnoses, radiologists should be aware of the range of pathology that can affect the spinous process.
